# Recombinant Atrial Natriuretic Peptide Prevents Aberrant Ca^2+^ Leakage through the Ryanodine Receptor by Suppressing Mitochondrial Reactive Oxygen Species Production Induced by Isoproterenol in Failing Cardiomyocytes

**DOI:** 10.1371/journal.pone.0163250

**Published:** 2016-09-22

**Authors:** Wakako Murakami, Shigeki Kobayashi, Takehisa Susa, Takuma Nanno, Hironori Ishiguchi, Takeki Myoren, Shigehiko Nishimura, Takayoshi Kato, Akihiro Hino, Tetsuro Oda, Shinichi Okuda, Takeshi Yamamoto, Masafumi Yano

**Affiliations:** Division of Cardiology, Department of Medicine and Clinical Science, Yamaguchi University Graduate School of Medicine, 1-1-1 Minamikogushi, Ube, Yamaguchi 755–8505, Japan; Western University, CANADA

## Abstract

Catecholamines induce intracellular reactive oxygen species (ROS), thus enhancing diastolic Ca^2+^ leakage through the ryanodine receptor during heart failure (HF). However, little is known regarding the effect of atrial natriuretic peptide (ANP) on ROS generation and Ca^2+^ handling in failing cardiomyocytes. The aim of the present study was to clarify the mechanism by which an exogenous ANP exerts cardioprotective effects during HF. Cardiomyocytes were isolated from the left ventricles of a canine tachycardia-induced HF model and sham-operated vehicle controls. The degree of mitochondrial oxidized DNA was evaluated by double immunohistochemical (IHC) staining using an anti-VDAC antibody for the mitochondria and an anti-8-hydroxy-2′-deoxyguanosine antibody for oxidized DNA. The effect of ANP on ROS was investigated using 2,7-dichlorofluorescin diacetate, diastolic Ca^2+^ sparks assessed by confocal microscopy using Fluo 4-AM, and the survival rate of myocytes after 48 h. The double IHC study revealed that isoproterenol (ISO) markedly increased oxidized DNA in the mitochondria in HF and that the ISO-induced DNA damage was markedly inhibited by the co-presence of ANP. ROS production and Ca^2+^ spark frequency (CaSF) were increased in HF compared to normal controls, and were further increased in the presence of ISO. Notably, ANP significantly suppressed both ISO-induced ROS and CaSF without changing sarcoplasmic reticulum Ca^2+^ content in HF (p<0.01, respectively). The survival rate after 48 h in HF was significantly decreased in the presence of ISO compared with baseline (p<0.01), whereas it was significantly improved by the co-presence of ANP (p<0.01). Together, our results suggest that ANP strongly suppresses ISO-induced mitochondrial ROS generation, which might correct aberrant diastolic Ca^2+^ sparks, eventually contributing to the improvement of cardiomyocyte survival in HF.

## Introduction

β-adrenal stimulation has been consistently demonstrated to induce cardiomyocyte injury even in normal cardiomyocytes [1–3]. For example, Mann et al. [1] reported that catecholamines induced the c-AMP-dependent intracellular Ca^2+^ overload of normal cardiomyocytes, subsequently leading to cardiomyocyte dysfunction and cardiomyocyte injury such as contraction band necrosis and apoptosis. Bovo et al. [3] further reported that excess β-adrenal stimulation caused abnormal elevation of mitochondrial reactive oxygen species (ROS), leading to the generation of arrhythmogenic Ca^2+^ waves in normal cardiomyocytes of the rabbit ventricle. Catecholamine-induced Ca^2+^ overload, in turn, damaged the intracellular mitochondria, resulting in enhancing mitochondrial ROS production [3–5]. In failing cardiomyocytes, on the other hand, spontaneous diastolic Ca^2+^ leakage from the ryanodine receptor (RyR2) was shown to occur irrespective of excess catecholamines, leading to intracellular Ca^2+^ overload and depletion of sarcoplasmic reticulum (SR) Ca^2+^ content, resulting in enhanced cardiomyocyte dysfunction and arrhythmogenicity [6–10]. Furthermore, even low dose catecholamines and phosphodiesterase (PDE) III inhibitors markedly enhance the diastolic Ca^2+^ leakage from RyR2 as compared with normal cardiomyocytes [6–11].

Atrial natriuretic peptide (ANP) is released from the atrium by mechanical stimulation [12], and serum ANP levels are increased in patients with heart failure (HF) [13]. Hayashi et al. [14] reported that an exogenous ANP, carperitide, has an anti-renin-angiotensin-aldosterone system effect and an anti-catecholamine effect in patients with HF. ANPs, which bind to the membrane guanylate coupled A receptor (GCA-R) that is distributed in vascular smooth muscle, the endothelium, central and peripheral nervous system, adrenals, kidney, spleen, and heart, consist of multifunctional biologically active peptides [15]. Although recent advances have led to a better understanding of the functions of ANP/GCA-R binding including vasodilation and diuretic, anti-oxidative, anti-catecholaminergic, and anti-apoptotic effects [15, 16], little is known about the direct molecular mechanisms toward failing cardiomyocytes. Here, we clarified the mechanism by which an exogenous ANP exerts cardioprotective effects in failing cardiomyocytes.

## Methods

### Canine heart failure model induced by rapid right ventricular pacing

All dogs used in the present study were female beagles (10–13 kg body weight, 3–4 years old) obtained from Kitayama Labes Co., Ltd. (Ina, Japan). The housing and husbandry conditions at the Science Research Center at Yamaguchi University were as follows. Housing: a large separate cage (90D × 85W × 80H cm) was provided for each dog (12 cages in total). Husbandry conditions: light/dark cycle (12 h/12 h) 7 am–7 pm; temperature 21°C ± 1°C; food: food (TC-2, Oriental Yeast Co., Ltd., Tokyo, Japan) was provided every day; water: drinking water, ad libitum. Health checks were performed daily both before and after pacemaker implantation by the staff in both the operated group and the sham operated group. If necessary, the dogs were assessed and treated by veterinarians.

In 10 adult beagles, HF was induced by the continuous application of rapid right ventricular pacing at 250 bpm using an externally programmable miniature pacemaker (Medtronic Inc., Minneapolis, MN, USA or Taisho Biomed Instruments Co., Ltd., Osaka, Japan) for 28 days, as described previously [6–8, 17]. The dogs were deeply anaesthetized with isoflurane and an intravenous injection of sodium pentobarbital (50 mg/kg) so that a pacemaker lead could be inserted into the right ventricle apex via the left jugular vein under fluoroscopy and connected to a pacemaker implanted subcutaneously in the neck. We utilized 10 sham operated dogs as controls.

### Hemodynamic measurements by echocardiography

Before isolation of cardiomyocytes from LVs of the sham operated controls and the paced dogs at 4 weeks, we measured the heart rate, blood pressure, and indices of cardiac function by echocardiography to confirm that pacing over the 4-week period induced HF under conscious condition. At the end of the study, the dogs were euthanized with isoflurane and an intravenous injection of sodium pentobarbital and ventilated mechanically, followed by rapid removal of the heart as previous described [6–8, 17]. The hearts were rapidly excised via thoracotomy. These procedures were performed in an animal surgery room of the Science Research Center at Yamaguchi University. This study conforms to the Guide for the Care and Use of Laboratory Animals published by the US National Institutes of Health (NIH Publication No. 85–23, revised 1996). All animal protocols were approved by the Yamaguchi University School of Medicine Animal Experiment Committee (institutional permission # 23–027).

### Isolation of cardiomyocytes

Cardiomyocytes were isolated from the left ventricle (LV) free wall of the beagles as described previously with some modification [6–8, 17]. Briefly, a wedge of the LV free wall perfused by a diagonal branch of the left anterior descending coronary artery was resected from the whole heart and quickly perfused with perfusion buffer (PB) (95% O_2_/5% CO_2_-bubbled minimal essential medium (Sigma, St. Louis, MO, USA), 10 mM taurine, 4 mM creatinine, 2 mM glutamax, 24 mM HEPES, 30 mM 2,3-butanedione monoxine, PH 7.3). Then, antegrade perfusion from the coronary artery branch was performed for 1 h with perfusion buffer plus collagenase (95% O_2_/5% CO_2_-bubbled minimal essential medium supplemented with 50 μM Ca^2+^, 0.5 mg/mL collagenase B, 0.5 mg/mL collagenase D, and 0.02 mg/mL protease type XIV). The temperature of the perfusion buffer was maintained at 37°C. Finally, the perfused LV was minced with scissors and rod-shaped adult canine cardiomyocytes were prepared as follows. The Ca^2+^ concentration in the incubation medium was gradually increased to a final concentration of 1 mM (50 μM, 125 μM, 300 μM, and 1 mM). The isolated cardiomyocytes were transferred to laminin-coated glass culture dishes at 37°C in a 95%O2/5%CO2 atmosphere for 1 h. Culture medium in the culture dishes was composed of equal volumes of the incubation buffer (the PB containing 5% bovine serum albumin and 0.1 mM Ca^2+^) and the free-serum medium (DMEM, 24 mM HEPES, 6 mM taurine, 5 mM creatine, 2 mM L-carnitine, 100 U/mL penicillin, and 100 μg/mL streptomycin). After attachment of cardiomyocytes on the bottom of culture dishes, the culture medium was exchange one time, and then we kept each culture dish at 37°C in the incubator until starting the following experiments, as described previously [8, 11,17].

### Measurement of the effect of ANP antioxidative stress on intact cardiomyocytes using 2,7-dichlorofluorescin diacetate (DCFH-DA)

The fluorescent probe DCFH-DA (Molecular Probes, Eugene, OR, USA), was used to assess intracellular ROS formation in canine cardiomyocytes, as described previously [7, 11, 18]. Fluorescence images (excitation at 490 nm, emission at 530 nm) were acquired using a laser scanning LSM 510 confocal microscope (Carl Zeiss, Oberkochen, Germany).

### Measurement of mitochondrial oxidative stress by dual immunohistochemistry (IHC)

The level of mitochondrial oxidized DNA was evaluated by double IHC staining using an anti-VDAC antibody to detect the mitochondria and an anti-8-hydroxy-2′-deoxyguanosine (8-OHdG) antibody for the oxidized DNA as previously described, with slight modification [19]: after cardiomyocyte isolation, the cells were fixed with 2% paraformaldehyde for 10 min. Next, they was incubated with an RNase solution (10 mg/mL RNase A in 5 mM Tris-HCl (pH7.5) and 7.5 mM NaCl) for 60 min at 37°C and then incubated with blocking solution including 0.05% Triton X-100 (50 μL Triton, 0.1 g bovine serum albumin in 10 mL phosphate buffered saline) for 30 min. After washing-out of the blocking solution, the cells were incubated with 1:100 diluted anti-VDAC antibody (rabbit polyclonal antibody to VDAC1/Porin, Abcam, Cambridge, UK) and 1:100 diluted anti 8-OHdG antibody (mouse monoclonal antibody (clone N451, JaICA, Tokyo) overnight at 4°C. After washing away unbound primary antibodies, 1:300 diluted Alexa Fluor 488 rabbit anti-mouse IgG (Invitrogen, Carlsbad, CA, USA) and 1:300 diluted Alexa Fluor 633 goat anti-rabbit IgG (Invitrogen) were used as secondary antibodies to develop color imaging. After washing, the nuclei were stained with 1:800 diluted DAPI. After 5 min following washing out the DAPI solution, the cardiomyocytes were encased with Vectashield (Vector Laboratories, Burlingame, CA, USA). Finally, the stained cells were imaged 405/488/633 nm excitation using confocal microscopy (LSM510 META). We then quantified the density of 8-OHdG / mitochondria (VDAC) density through the densitometric measurements of 8-OHdG and VDAC fluorescence intensities, and (fluorescence intensity of 8-OHdG) / (fluorescence intensity of VDAC) was defined as “relative mitochondrial DNA damage”.

### Analysis of Ca^2+^ sparks using laser scanning confocal microscopy

After isolation of cardiomyocytes from LV, Ca^2+^ sparks were measured as previously described [8, 11, 17] using the Zeiss-LSM5 laser scanning confocal microscope equipped with an argon ion laser and coupled to an inverted microscope (Axiovert 100, Carl Zeiss) with a Zeiss 4× oil-immersion Plan-Neofluor objective (1.3 numerical aperture; excitation at 488 nm; emission > 505 nm). The cardiomyocytes were loaded with 20 μM Fluo-4 AM (Molecular Probes) for 20 min at room temperature in the dark and then washed. Within 30 s after the start of pacing, the intracellular Ca^2+^ transient amplitudes reached the steady state. Therefore, Ca^2+^ sparks were recorded from 30–50 s after the start of pacing at the rate of 0.5 Hz. The Ca^2+^ spark frequency (CaSF) for each image (also for each group) was measured in the same scanning window to exclude the possibility of different CaSF caused by different laser scanning times. Each cardiomyocyte was scanned repeatedly at 325.7 Hz along a line parallel to the longitudinal axis of the cell to avoid the nucleus. The data were analyzed with SparkMaster, an automated analysis program that allows rapid and reliable Ca^2+^ spark analysis in confocal line-scan images, as described previously [8, 11, 17]. The measurements of Ca^2+^ spark assay experiments were completed within 3 h to avoid time-dependent changes of cardiomyocyte function.

### Measurement of intra-sarcoplasmic reticulum Ca^2+^ concentrations in cardiomyocytes

A caffeine-induced Ca^2+^ transient current was measured by first applying a stimulation train at 0.5 Hz for 60 s and then rapidly switching the superfusion solution to a solution containing 20 mM caffeine for 5–6 s, as previously described [8, 11, 17].

### Effect of isoproterenol (ISO) and/or ANP on cardiomyocyte cell survival

After isolation of cardiomyocytes from LV, the cells were incubated in the culture dish containing the culture medium [equal volumes of the incubation buffer (the PB containing 5% bovine serum albumin and 0.1 mM Ca^2+^) and the free-serum medium (DMEM, 24 mM HEPES, 6 mM taurine, 5 mM creatine, 2 mM L-carnitine, 100 U/mL penicillin, and 100 μg/mL streptomycin)] in the presence of distilled water (baseline), ANP (10 nM), ISO (10 nM), or ANP+ISO, respectively. The isolated cardiomyocytes in the culture dish were initially paced for 2 min in order to confirm which cells were cardiomyocytes and viable by observing the cell shortening on video, and then survival rate of the cardiomyocytes in each culture dish was calculated at 0 h, 24 h and 48 h. Isolated cardiomyocytes were seeded in the culture dishes with grids and at least 200 cells / 4 mm^2^ were attached to the bottom of the dish. Viable cardiomyocytes were defined as those with a rod shaped and striations [20, 21].

To examine whether our serial subjective visual determination for evaluation of viable cardiomyocytes is accurate or not, we evaluated accurate rates of viable cardiomyocytes with our serial subjective visual determination in comparison with a quantitative evaluation with trypan blue staining at 0 h, 24 h, and 48 h, respectively. The Accuracy rates at 0 h, 24 h, 48 h were 2282 cells/2312 cells (98.7%), 2161 cells/2211 cells (97.7%), and 2173 cells/2290 cells (94.9%), respectively, when rod shaped cells unstained with trypan blue, were defined as true alive cardiomyocytes (S1 Fig).

### Statistical analysis

Comparisons across ISO(+/-), ANP(+/-), and HF(+/-) were independently verified with multivariate analysis of variance in the experimental studies. A Kruskal Wallis ANOVA was used to evaluate the antioxidative effect of ANP on intact cardiomyocytes. All analyses were performed using SPSS 18.0 software (SPSS Inc., Chicago, IL, USA). P values less than 0.05 were considered statistically significant. All values are expressed as means ± SE.

## Results

### Comparison of hemodynamics between sham hearts and the HF model

After 4 weeks of rapid pacing, a decreased LV fractional shortening (FS), dilated LV end-diastolic dimension (LVDD), and dilated LV end-systolic dimension (LVDS) were confirmed in the HF group as compared with sham operated controls (FS; 34.9 ± 1.6% in Sham vs 13.7 ± 1.1% in HF, P<0.0001; LVDD; 31.6 ± 0.4 mm in Sham vs 40.0 ± 0.5 mm in HF, P<0.0001; LVDS; 20.6 ± 0.6 mm in Sham vs 34.6 ± 0.8 mm in HF, P<0.0001) (Table 1). No difference was observed in heart rate (HR) and systolic blood pressure (BPs) between the HF group and sham operated controls. These data were compatible with the hemodynamic data that were previously reported for this model [8, 11, 22].

**Table 1:** Hemodynamics data.

	HR(bpm)	SBP(mmHg)	LVDD(mm)	LVDS(mm)	LVFS(%)
Sham(n = 10)	130±4	125±5	31.6±0.4	20.6±0.6	34.9±1.6
HF(n = 10)	132±4	122±4	40.0±0.5*	34.6±0.8*	13.7±1.1*

Sham, sham operated control; HF, pacing-induced heart failure group; HR, heart rate; SBP, systolic blood pressure; LVDD, left ventricular end-diastolic diameter; LVDS, left ventricular end-systolic diameter; LVFS, left ventricular fractional shortening. Each point represents the means ± SE. The number of experiments is shown in parentheses. An unpaired T-test was employed to determine the statistical significance of the data (*p* value).

*p<0.0001 vs. Sham

### ANP anti-oxidative effect on intact cardiomyocytes

**Fig 1:**
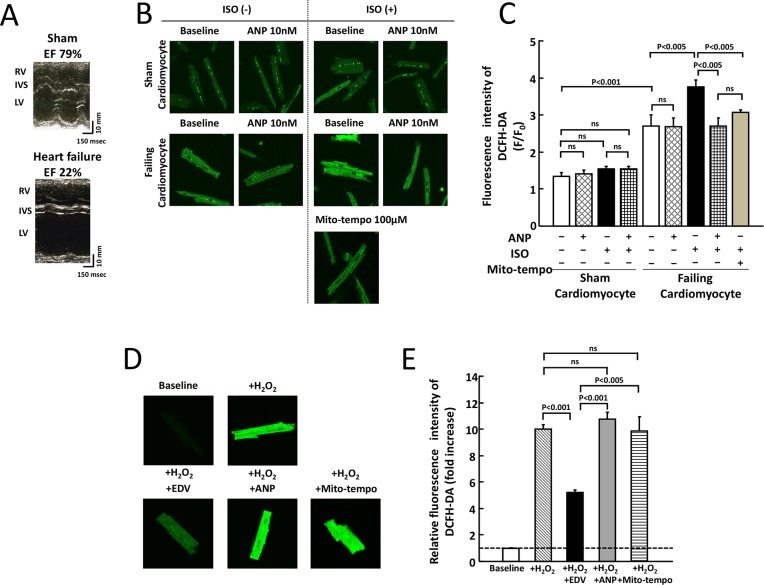
Effects of ISO and/or ANP on ROS production in sham and failing cardiomyocytes. A. Representative images of echocardiography in a sham operated dog and a HF dog. Left ventricular ejection fraction (LVEF) in a sham operated dog was 79%, while LVEF in a HF dog was 22%. RV, right ventricle; IVS, interventricular septum; LV, left ventricle; Sham, sham operated control. B. Representative images depicting intracellular ROS production in sham and failing cardiomyocytes corresponding to Fig A. Cardiomyocytes were subjected to immunofluorescence staining with a ROS-sensitive fluorescent dye (DCFH-DA) after electrical pacing at 0.5 Hz. Upper panels: sham cardiomyocytes. Bottom panels: failing cardiomyocytes. C. Bar graph representation of the data in Fig 1B. The bars indicate the means ± SE. Each group included 20–30 cells. At least 4 cells were evaluated for each preparation. D. Representative images depicting the antioxidant effect of the free radical scavenger edaravone (100 μM), ANP (10 nM) and Mito-tempo (100 μM) after exposure to H_2_O_2_ (25 μM) in sham cardiomyocytes. E. Bar graph representation of the data in Fig 1D. The bars indicate the means ± SE. Changes in the fluorescence intensities of DCFH-DA were compared among cell treatment with edaravone (100 μM), ANP (10 nM) and Mito-tempo (100 μM) Each group included 20–30 cells. At least 4 cells were evaluated for each preparation.

In the present study, we examined the anti-oxidant effect of ANP at concentration of 1 × 10^−6^_,_ 1 × 10^−7^, 1 × 10^−8^, and 1 × 10^−9^ M on ISO-induced intracellular ROS in failing cardiomyocytes. We found that the anti-oxidant action of ANP was maximized at 10^−8^ M and that there was no further effect at higher concentrations. Therefore, we performed the experiments at a concentration of 1 × 10^−8^ M, which corresponds to clinically used. Fig 1A shows representative images of echocardiography in a sham operated dog and a HF dog. Left ventricular ejection fraction (LVEF) in a sham operated dog was 79%, while LVEF in a HF dog was 22%. Sham cardiomyocytes and failing cardiomyocytes in Fig 1B were isolated from LVs of the sham operated dog and the HF dog in Fig 1A. Fig 1B shows representative fluorescence images after the application of a fluorescent probe for intracellular ROS, DCFH-DA (1 μM) to sham or failing cardiomyocytes. In sham cardiomyocytes, the level of DCFH-DA fluorescence intensity was not changed in the presence of ISO, ANP, or ISO + ANP as compared with that of the sham baseline (Fig 1B and 1C). On the other hand, the intracellular ROS at baseline in failing myocytes was increased compared with that at baseline in sham cardiomyocytes and, furthermore, the level of intracellular ROS was markedly increased by the application of low dose ISO. Notably, ANP significantly suppressed ISO-induced ROS production like as Mito-tempo (Fig 1B and 1C). In sham cardiomyocytes, the fluorescence intensity was markedly increased after the addition of 25 μM H_2_O_2_, whereas it was restored to approximately normal levels in the presence of 100 μM edaravone, which is a free radical scavenger. In contrast, the fluorescence intensity was not altered in the presence of 10 nM ANP or 100 μM Mito-tempo (Fig 1D and 1E).

**Fig 2:**
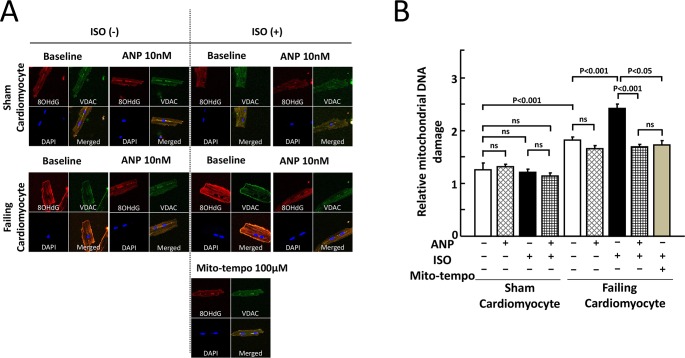
Effects of ISO and/or ANP on mitochondrial oxidative stress in sham and failing cardiomyocytes. A. Double IHC using an anti-VDAC antibody for the mitochondria and an anti-8-OHdG antibody for oxidized DNA is shown. Nuclei were counterstained with DAPI. Cytoplasmic 8-OHdG immunoreactivities were co-localized with mitochondrial protein VDAC immunoreactivities. Upper panels: sham cardiomyocytes. Middle panels: failing cardiomyocytes. Bottom panels: failing cardiomyocytes exposed to ISO (10 nM) and the mitochondria-targeted ROS scavenger Mito-tempo (100 μM). B. Bar graph representation of the data in Fig 2A. Relative mitochondrial DNA damage = (fluorescence intensity of 8-OHdG) / (fluorescence intensity of VDAC). The bars indicate the means ± SE. Each group included 20–30 cells. At least 6 cells were evaluated for each preparation.

Fig 2 shows the results of the double IHC study using an anti-VDAC antibody for the mitochondria and an anti-8-OHdG antibody for oxidized DNA. The mitochondrial distribution (green color) is restricted to the cytosol. The IHC staining using the anti-VDAC antibody is specific, and the IHC staining (red color) using the anti-8-OHdG antibody is very similar to that of the mitochondrial distribution (green color). The addition of ISO, ANP, or ISO + ANP had no appreciable effect in sham cardiomyocytes, whereas the addition of ISO markedly increased the detection of oxidized DNA in the mitochondria in failing cardiomyocytes. In addition, the ISO-induced mitochondrial ROS production was markedly inhibited by the co-presence of 10 nM ANP or 100 μM Mito-tempo, which is a mitochondria-targeted ROS scavenger (Fig 2A). Fig 2B showed that an increase in relative mitochondrial DNA damage induced by ISO was significantly decreased in co-presence of ANP or Mito-tempo in failing cardiomyocytes. These results suggested that ANP inhibited ISO-induced ROS production in mitochondria in failing cardiomyocytes.

### Effects of ISO and/or ANP on Ca^2+^ handling in isolated cardiomyocytes

**Fig 3:**
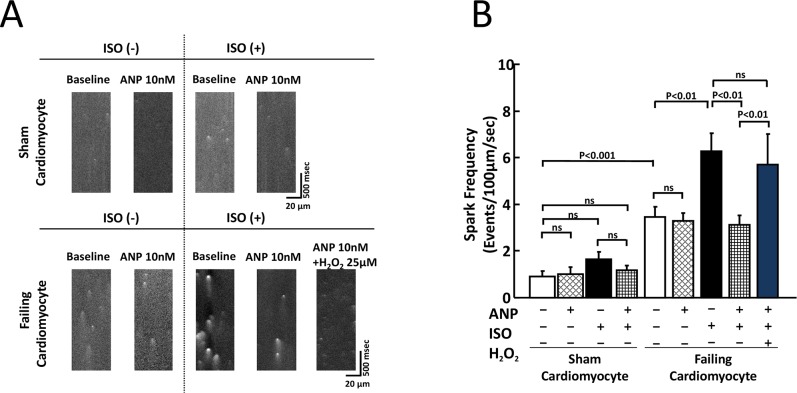
Effect of ISO or ANP on Ca^2+^ sparks in sham and failing cardiomyocytes. A. Representative data for diastolic Ca^2+^ sparks in sham and failing cardiomyocytes. B. Bar graph representation of the data in Fig 3A. The bars indicate the means ± SE. Each group included 20–30 cells. At least 4 cells were evaluated for each preparation. Notably, ISO-induced aberrant diastolic Ca^2+^ sparks were inhibited by 10 nM ANP, but upon the addition of H_2_O_2_ (25 μM), aberrant diastolic Ca^2+^ sparks reappeared in the failing cardiomyocytes.

The addition of ISO at a concentration of 10 nM or below did not have any appreciable effect on CaSF in sham cardiomyocytes; however, the addition of ISO at a concentration of 30 nM or above significantly increased CaSF (S2 Fig). There was no difference in CaSF (events/100μm/sec) between 0 nM ISO and 10 nM ISO (0.90 ± 0.25 vs 1.66 ± 0.30, p = 0.07), while there was significant difference in CaSF (events/100μm/sec) between 0 nM ISO and 30 nM ISO (0.90 ± 0.25 vs 3.85 ± 0.66, P = 0.0002), as shown in S2 Fig. On the basis of these results, we defined 10 nM ISO as the “low dose.” As shown in Fig 3A and 3B, the addition of 10 nM ISO and/or ANP did not have any appreciable effect on CaSF in sham cardiomyocytes. In failing cardiomyocytes, the CaSF at baseline was significantly increased as compared with sham cardiomyocytes. The addition of 10 nM ISO to failing cardiomyocytes significantly increased the CaSF. Notably, the co-addition of ISO and ANP to failing cardiomyocytes largely decreased the level of ISO-enhanced CaSF. Upon the addition of 25 μM H_2_O_2_ to the co-presence of ISO and ANP, CaSF was significantly increased.

**Fig 4:**
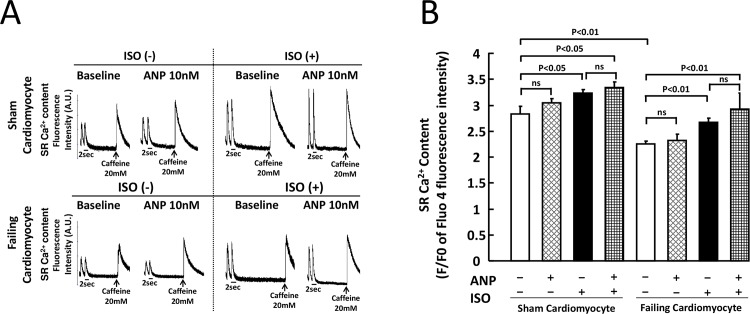
SR Ca^2+^ content in sham and failing cardiomyocytes. A. Measurement of SR Ca^2+^ content by caffeine application. After isolation of cardiomyocytes, cardiomyocytes were loaded with 20 μM Fluo-4 AM (Molecular Probes) for 30 min at room temperature in the dark. Then, these cardiomyocytes were washed with Tyrode solution containing final concentration of 2 mM Ca^2+^. The cardiomyocytes were electrically stimulated by a field stimulator (IonOptix, MA) at a frequency of 0.5 Hz for 30 sec, and then final concentration of 20 mM caffeine were added. An arrow shows a point of addition of caffeine to the dish. B. Bar graph representation of the data in Fig 4A. Each group included 20–30 cells. At least 4 cells were evaluated for each preparation. The bars indicate the means ± SE.

The SR Ca^2+^ content data of the corresponding groups are shown in Fig 4A and 4B. In sham cardiomyocytes, ISO application significantly increased [Ca^2+^]_SR_ irrespective of the presence of ANP (Baseline vs ISO, p<0.05). In failing cardiomyocytes, [Ca^2+^]_SR_ at baseline was significantly decreased as compared with sham cardiomyocytes. [Ca^2+^]_SR_ was increased by the addition of ISO to failing cardiomyocytes, although there no difference was noted following ANP addition (Fig 4A and 4B).

### Effect of ISO and/or ANP on cardiomyocyte survival

In cultured cardiomyocytes, a normal rod shape was observed at the start of the experiment (n = 1804, 1540 in sham and failing cardiomyocytes, respectively). Following the addition of ANP (10 nM), ISO (10 nM), or ANP (10 nM) + ISO (10 nM), the cells showed a morphological change within 48 h or preserved their smooth-surfaced rod shape throughout the time period. Some cells showed a shortening of the cellular long-axial diameter, followed by maximal shrinkage to become almost completely round with a rough surface, along with multiple budding formations. We judged cardiomyocyte viability based on cell appearance to determine the survival rate.

**Fig 5:**
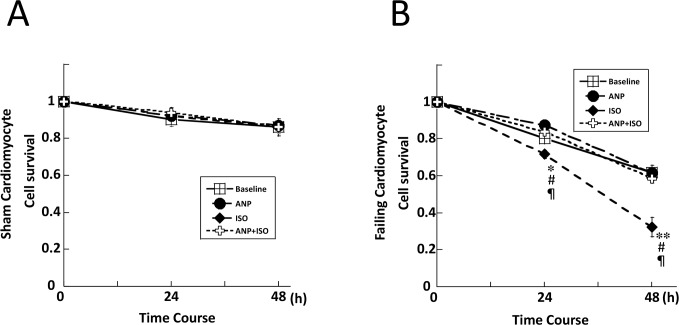
Effects of ISO and/or ANP on cell survival in sham and failing cardiomyocytes. *p<0.05 vs. failure (baseline), **p<0.01 vs. failure (baseline), ^#^*p*<0.05 vs. failure (ANP), ^¶^*p*<0.05 vs. failure (ANP+ISO). The bars indicate the means ± SE.

In sham cardiomyocytes, the baseline cell survival rate was decreased to 90% at 48 h after incubation but there was no difference in the cell survival among the baseline, ANP, ISO, or ANP + ISO groups at 0 h, 24 h, and 48 h culture (Fig 5A). On the other hand, in failing cardiomyocytes, the cell survival in baseline and following ANP treatment gradually decreased to 59% (Fig 5B). However, the survival rate in failing myocytes was significantly lower in the presence of ISO whereas it was significantly improved by co-incubation of ISO + ANP (ISO vs. ISO + ANP, 33 ± 5% vs. 56 ± 2%, P < 0.01) (Fig 5B).

## Discussion

To our knowledge, this study represents the first demonstration that even low dose of the synthetic catecholamine, ISO, which does not cause injury in sham cardiomyocytes, induced both an increase of intracellular ROS and of diastolic Ca^2+^ release through RyR2, leading to decreased cell survival in failing cardiomyocytes. Furthermore, an important molecular mechanism by which ANP prevents ISO-induced cardiomyocyte injury in failing cardiomyocytes is the inhibition of ISO-induced mitochondrial oxidative stress by ANP-induced GCA-R signal transduction. This mechanism was supported by several lines of experimental findings, as described below.

The measurement of intracellular ROS using a DCFH-DA assay showed that the baseline level of intracellular ROS in failing myocytes was increased as compared with that at baseline in sham cardiomyocytes; furthermore, the level of intracellular ROS was markedly increased in the former cells by an application of low dose ISO. Notably, ANP significantly suppressed the ISO-induced ROS production. Similarly, double IHC analysis revealed that ISO markedly increased the level of oxidized DNA in mitochondria during HF, and the ISO-induced DNA damage was markedly inhibited by the co-presence of ANP. Furthermore, the baseline CaSF was also increased in HF, and this level was markedly increased in the presence of ISO. Notably, in HF, ANP significantly suppressed ISO-induced ROS and CaSF. Accordingly, the cell survival rate in HF was significantly decreased in the presence of ISO, whereas it was significantly increased by the co-presence of the ANP like compound Mito-tempo.

Although the precise mechanism underlying the antioxidant action of ANP is still unknown, immune response [23–25] and the suppression of the renin-angiotensin-aldosterone system and sympathetic nerve activity have been suggested [14, 16, 26–28]. In this study, the demonstration that ISO-induced mitochondrial oxidative stress was inhibited by exogenous ANP in a HF model suggests that the primary site of ANP antioxidant action is the mitochondria. Myocardial oxidative stress has been shown to be elevated in failing cardiomyocytes [7, 18, 29, 30] and the main source of such stress is thought to be from the mitochondria themselves in the failing cardiomyocytes [31, 32]. In the present study, even low dose ISO markedly increased mitochondrial ROS; furthermore, exogenous ANP inhibited the ROS production from the mitochondria in the failing cardiomyocytes.

With regard to the mechanism how ANP suppressed ISO-induced mitochondrial ROS generation, for which we have no evidence in the present study, the beneficial effects of ANP might be due to preserved mitochondrial biogenesis which is significantly affected in pathophysiological conditions such as HF and hypertrophy [33] and/or reduced mitochondrial ROS-induced mitogen-activated protein kinase (MAPK) signaling as shown in phenylephrine-treated cardiomyocytes [34]. Adrenergic receptor stimulation enhances Na^+^/H^+^ exchanger isoform 1 (NHE-1) activity under such pathophysiological conditions. In turn, the resultant increase in intracellular Na^+^ concentrations increases cytosolic Ca^2+^ levels via Na^+^-Ca^2+^ exchanger. The increased cytosolic Ca^2+^ is taken up by mitochondria through Ca^2+^ uniporter, resulting in an elevated Ca^2+^ levels in mitochondria. Eventually, the mitochondrial ROS generation accompanied by Ca^2+^ overload in mitochondria causes hypertrophy via MAPK signaling [33,34]. ANP/cyclic GMP signal can also contribute to reduce the cardiomyocyte growth by inhibition of the NHE-1 activity under such pathophysiology conditions [35]. Taken together, the ROS generation in mitochondria might be suppressed by ANP-induced NHE-1 inhibition in HF.

Another mechanism by which ANP improved cell survival in failing cardiomyocytes may be contributed to protective actions on cardiomyocyte growth and contractile function via ANP/cyclic GMP-dependent protein kinase-I (PKG-I) signaling [36–38]. ANP binds to GCA-R, which then catalyzes the synthesis of cGMP, leading to the activation of PKG. The PKG signaling phosphorylates specific target proteins at sarcolemma, such as the regulator of signaling 2 and transient receptor potential canonical-6 Ca^2+^ channels, which, in turn, moderates the adverse hypertrophic growth response of cardiomyocytes to stressors, such as pressure overload and angiotensin II [36–38].

**Fig 6:**
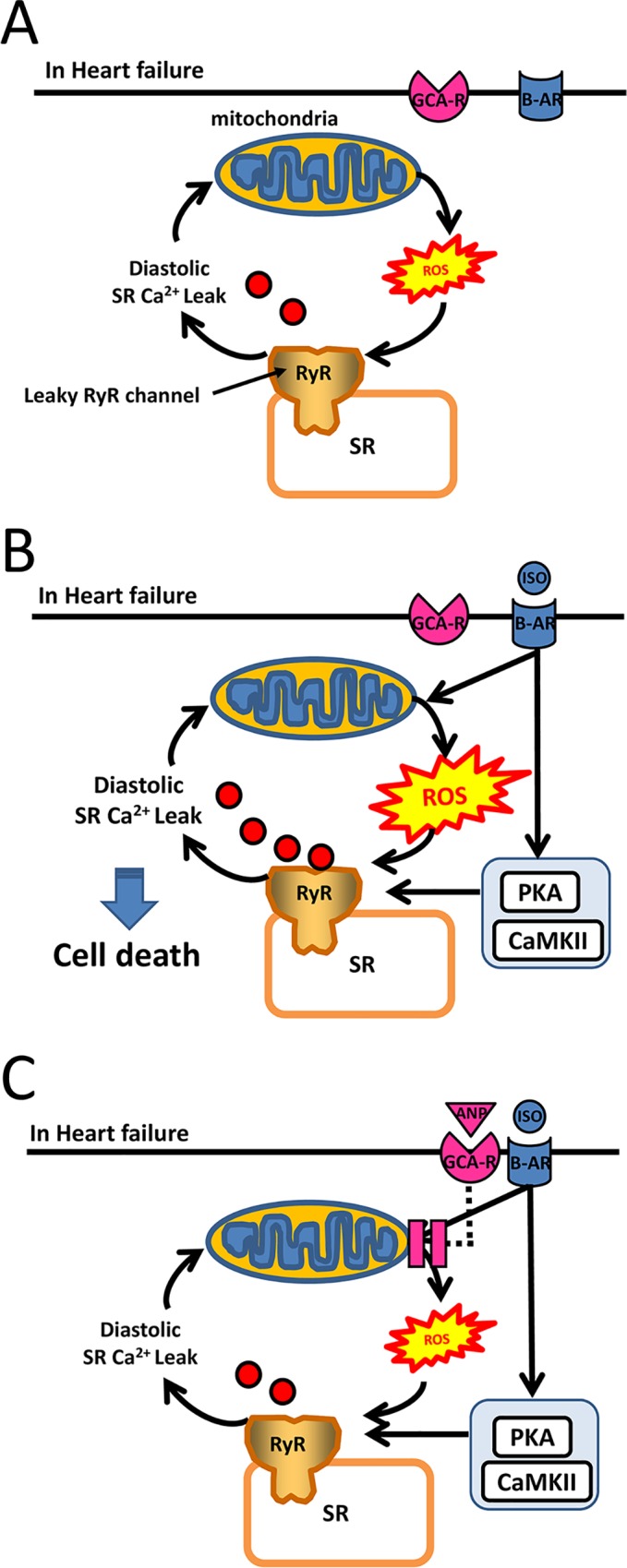
Proposed ANP cardioprotective mechanism in heart failure. A. Leaky RyR channel in failing cardiomyocytes. B. ISO enhanced diastolic SR Ca^2+^ leak. C. ANP inhibited ISO-induced mitochondrial ROS, leading to the decrease of diastolic Ca^2+^ leak through RyR. ANP, atrial natriuretic peptide; GCA-R, membrane guanylate coupled A receptor; ISO, isoproterenol; β-AR, β adrenal receptor; PKA, protein kinase A; CaMKII, Ca^2+^/calmodulin-dependent protein kinase II; RyR, ryanodine receptor; ROS, reactive oxygen species; SR, sarcoplasmic reticulum.

On the basis of our results, we therefore propose the following model for the molecular basis of the cardioprotective action of ANP against ISO-induced myocardial damage (Fig 6). In failing cardiomyocytes, intracellular ROS is increased compared with that of normal cardiomyocytes (Fig 6A). ISO application enhances the already increased intracellular ROS production and diastolic Ca^2+^ release through RyR2 via two potential mechanisms (Fig 6B). One such mechanism represents Ca^2+^ overload-dependent mitochondrial ROS production, wherein β adrenergic stimulation causes c-AMP-dependent PKA activation. PKA then, to a large degree, phosphorylates Ser 16 of phospholamban and Ser 2808 of RyR2, which increases diastolic Ca^2+^ release through RyR2 [7, 11, 18]. Subsequently, the intracellular Ca^2+^ overload damages the mitochondria, resulting in increased ROS production [4]. The other mechanism is via Ca^2+^-independent mitochondrial ROS production during β adrenergic stimulation [3, 5, 39], which might be attributed to ISO-mediated Ca^2+^/calmodulin-dependent protein kinase II (CaMKII) activation [5]. The increased ROS then further enhances the aberrant Ca^2+^ release through the RyR2 [7, 18, 40]. Thus, a vicious circle is formed by mitochondrial ROS and diastolic Ca^2+^ release through RyR2. However, the ISO-induced ROS generation in the mitochondria and the diastolic Ca^2+^ release through RyR2 are markedly inhibited by the co-presence of ANP (Fig 6C). The antioxidant effects of ANP in failing cardiomyocytes thought to be secondary and result from other beneficial effects of ANP (e.g., NHE-1 inhibition), because the increase in intracellular ROS levels caused by H_2_O_2_ was not decreased by the co-presence of ANP (Fig 1D and 1E). Furthermore, upon the addition of H_2_O_2_ to co-presence of ISO and ANP, the CaSF was increased. This suggests that exogenous ROS directly enhances diastolic Ca^2+^ release through RyR2, consistent with previous reports [3]. In addition, ANP significantly inhibited ISO-induced CaSF in spite of an increase of SR Ca^2+^ content in cardiomyocytes (Fig 3B and Fig 4B). The reason might be stabilization of RyR2 due to the antioxidant effects of ANP [7, 18, 40]. Taken together, the suppression of ISO-induced ROS production by ANP would inhibit diastolic Ca^2+^ release through RyR2 in failing cardiomyocytes.

With regard to the mechanism relating to ISO-induced cell death, Ellison et al. reported that ISO (from 10 nM to 10 μM) caused cardiomyocyte death through phosphorylation of RyR2, which led to Ca^2+^ leakage from SR, and, in turn, cardiomyocyte apoptosis by activation of caspase, eventually cell death [41]. This is consistent with our data. We can presume that the phosphorylation of RyR2 via PKA and CaMKII activated by ISO is closely associated with intracellular Ca^2+^ handling abnormality, ROS production, and apoptosis process. In HF, even low dose of ISO might markedly enhance diastolic Ca^2+^ leakage through RyR2, because it phosphorylated the RyR2 via PKA and CaMKII [9–11].

The present study has several limitations. First, we examined ANP function on mitochondrial ROS production but did not clarify whether ANP affected NADPH oxidase and xanthine oxidase [42]. However, Bovo et al. [3] reported that high dose ISO (250 μM) induced mitochondrial ROS even in normal rabbit cardiomyocytes and that this was inhibited by Mito-tempo, a specific mitochondrial ROS inhibitor, but not by a xanthine oxidase inhibitor. In the present study, Mito-tempo and ANP similarly inhibited ISO-induced ROS, suggesting that the mitochondria played a critical role in the production of ROS by ISO in failing cardiomyocytes.

Second, with regard to the molecular mechanism of diastolic Ca^2+^ release through RyR2, Shannon et al. [2] reported that high dose ISO (250 nM) caused aberrant Ca^2+^ leakage by CaMKII activation in intact rabbit ventricular myocytes. This potential mechanism was not examined in our study. Although ISO (10 nM) significantly increased SR Ca^2+^ content in sham cardiomyocytes, the ISO did not increase CaSF. On the other hand, CaSF was significantly elevated with an increase of Ca^2+^ content in failing cardiomyocytes. These results may indicate that Ca^2+^ leakage through the failing RyR2 induced by low dose ISO is, to a large extent, caused by an increase in SR Ca^2+^ content.

Third, we did not show the direct action of ANP against mitochondrial oxidative stress. The technique using co-localization of VDAC and 8-OHdG could not demonstrate the mitochondria-specific oxidative damages. Therefore, we showed that ANP significantly suppressed ISO-induced mitochondrial ROS generation in comparison with Mito-tempo, a mitochondrially targeted antioxidant, in the present study. However, we need to carry out another techniques (e.g., MitoSOX, a mitochondrial specific fluorescence dye) in order to investigate whether ANP directly inhibited mitochondrial ROS generation.

## Conclusion

This study demonstrates that ANP strongly suppresses ISO-induced mitochondrial ROS generation, which might correct aberrant diastolic Ca^2+^ sparks and eventually contribute to the improvement of cardiomyocyte survival in HF.

## Supporting Information

S1 Fig Comparison of cardiomyocyte survival with a subjective visual determination with trypan blue staining. Firstly, numbers of cardiomyocytes were counted with our subjective visual determination (See methods section), and then the cardiomyocytes were exposed to 0.1% trypan blue dye (Sigma) for 5 min. After washing cardiomyocytes with the culture medium, the numbers of stained and unstained cells in the dishes were counted. Accurate rate of viable cardiomyocytes with the visual determination was calculated as follows: accurate rate (%) = 100 x (total rod shaped cells before trypan blue staining—stained rod shaped cells after trypan blue staining) / (total rod shaped cells before trypan blue staining). The independent experiments were carried out at 0 h, 24 h, 48 h, and viable cardiomyocytes within each square (4 mm^2^) surrounded with grids were counted. The Accuracy rates at 0 h, 24 h, 48 h were 2282 cells/2312 cells (98.7%), 2161 cells/2211 cells (97.7%), and 2173 cells/2290 cells (94.9%), respectively, when rod shaped cells unstained with trypan blue, were defined as true alive cardiomyocytes. Each group included more than 2100 cells. At least 600 cells were evaluated for each preparation. A bar indicates 200 μm long.
(TIF)

S2 Fig The effect of various concentrations of isoproterenol on Ca^2+^ spark frequency in sham cardiomyocytes. CaSF was measured in the presence of various concentrations of ISO (0, 3, 10, 30, 100 nM). Low dose of ISO (3 nM, 10 nM) did not increase CaSF as compared with 0 nM ISO, while 30 nM, 100 nM ISO significantly increased CaSF as compared with 0 nM ISO. Each group included 20–30 cells. At least 4 cells were evaluated for each preparation. The bars indicate the means ± SE. CaSF, frequency of Ca^2+^ sparks; ISO, isoproterenol
(TIF)

## Abbreviations

ANP: atrial natriuretic peptide
CaMK II: Ca^2+^/calmodulin-dependent protein kinase II
cAMP: 3'-5'-cyclic adenosine monophosphate
DCFH-DA: 2,7-dichlorofluorescin diacetate
HF: heart failure
ISO: isoproterenol
LV: left ventricular
8-OHdG: 8-hydroxy-2′-deoxyguanosine
PKA: protein kinase A
ROS: reactive oxygen species
RYR2: ryanodine receptor 2
CaSF: frequency of Ca^2+^ sparks
SR: sarcoplasmic reticulum
