# Supplementary figures and images for: Recombinant Atrial Natriuretic Peptide Prevents Aberrant Ca2+ Leakage through the Ryanodine Receptor by Suppressing Mitochondrial Reactive Oxygen Species Production Induced by Isoproterenol in Failing Cardiomyocytes
Source: PLoS One. 2016 Sep 22;11(9):e0163250. doi: 10.1371/journal.pone.0163250 (PMC5033569; doi:10.1371/journal.pone.0163250)

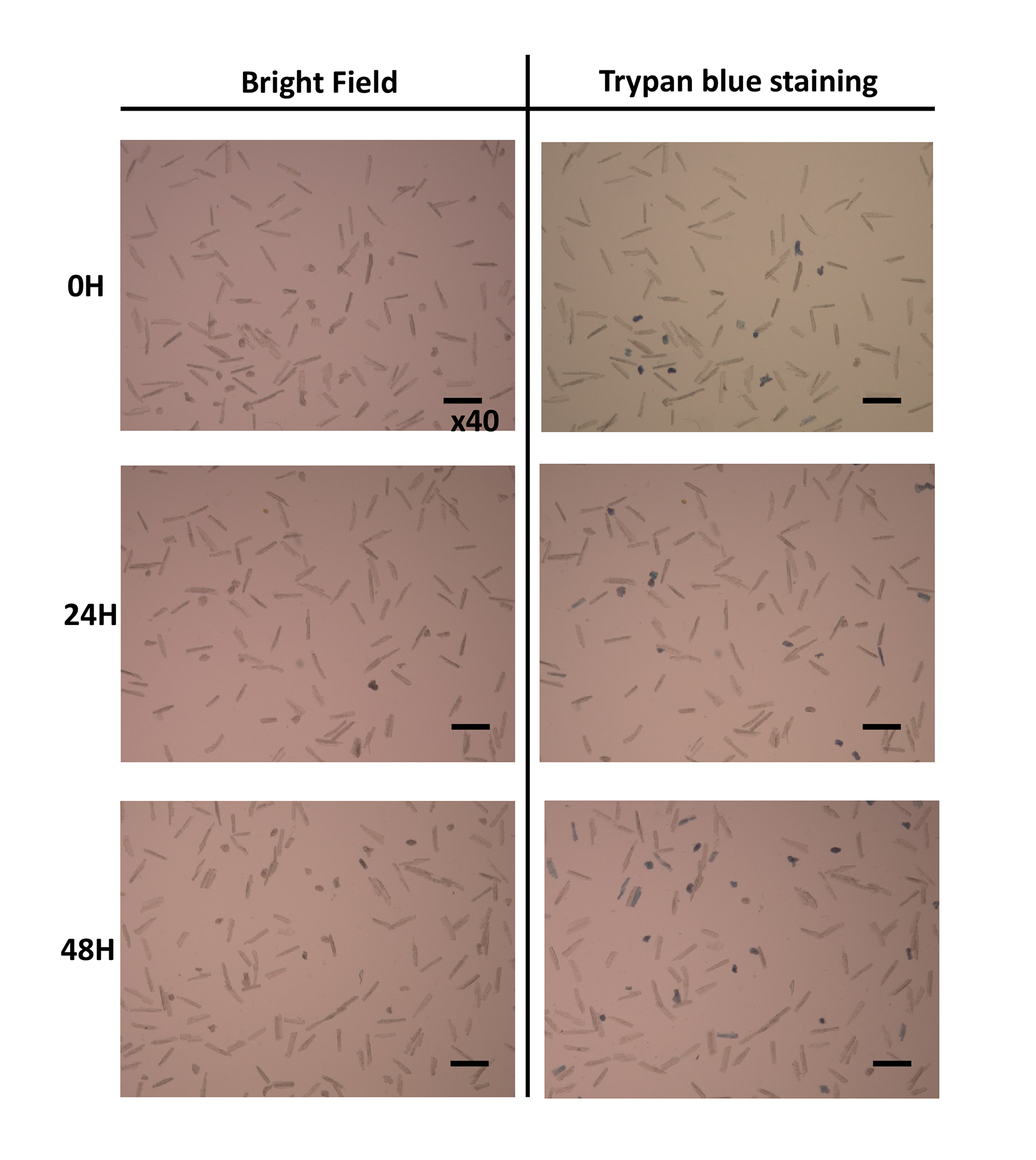

Supplement: S1 Fig — Firstly, numbers of cardiomyocytes were counted with our subjective visual determination (See methods section), and then the cardiomyocytes were exposed to 0.1% trypan blue dye (Sigma) for 5 min. After washing cardiomyocytes with the culture medium, the numbers of stained and unstained cells in the dishes were counted. Accurate rate of viable cardiomyocytes with the visual determination was calculated as follows: accurate rate (%) = 100 x (total rod shaped cells before trypan blue staining—stained rod shaped cells after trypan blue staining) / (total rod shaped cells before trypan blue staining). The independent experiments were carried out at 0 h, 24 h, 48 h, and viable cardiomyocytes within each square (4 mm2) surrounded with grids were counted. The Accuracy rates at 0 h, 24 h, 48 h were 2282 cells/2312 cells (98.7%), 2161 cells/2211 cells (97.7%), and 2173 cells/2290 cells (94.9%), respectively, when rod shaped cells unstained with trypan blue, were defined as true alive cardiomyocytes. Each group included more than 2100 cells. At least 600 cells were evaluated for each preparation. A bar indicates 200 μm long. (TIF) [file pone.0163250.s001.tif]

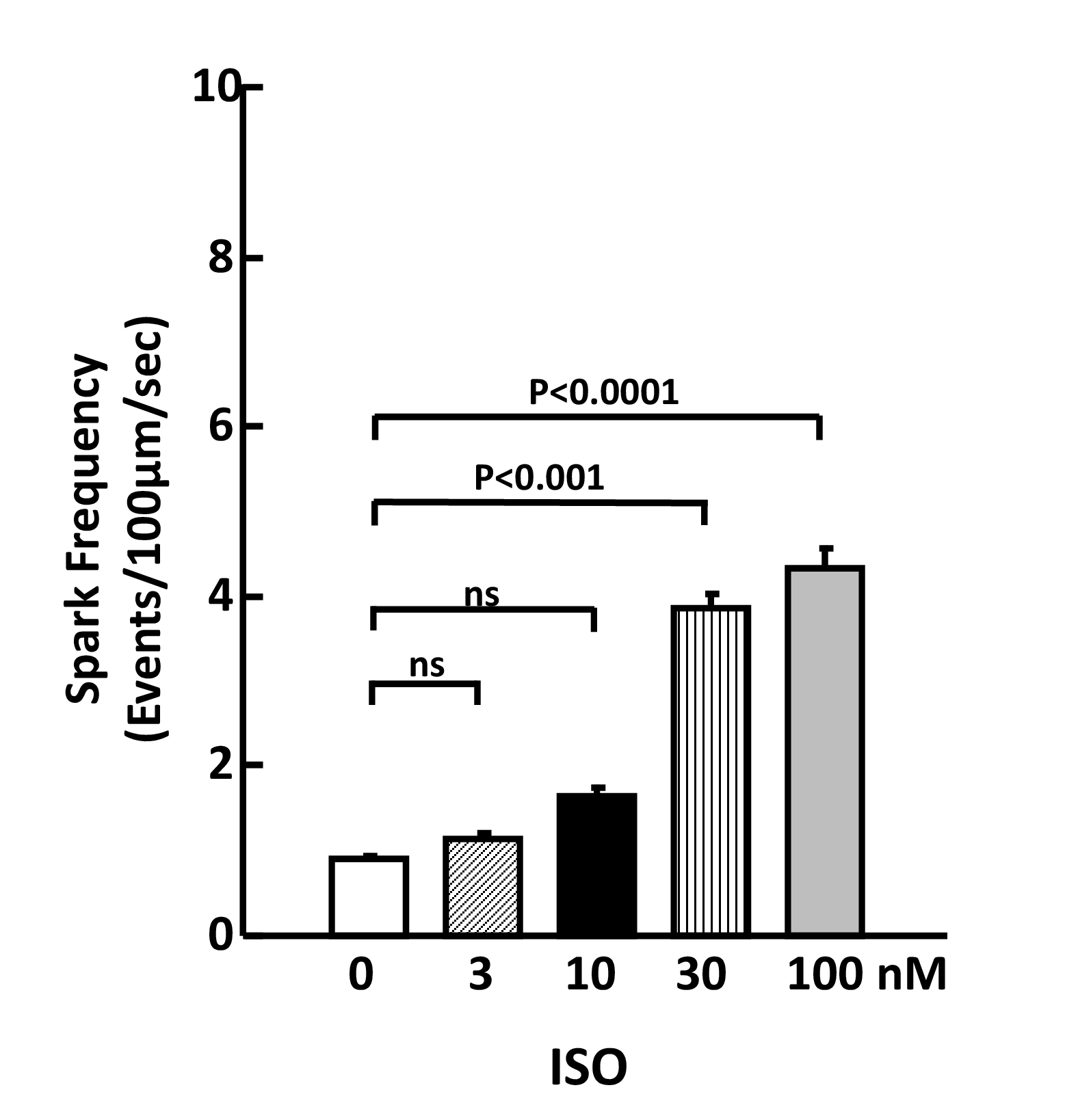

Supplement: S2 Fig — CaSF was measured in the presence of various concentrations of ISO (0, 3, 10, 30, 100 nM). Low dose of ISO (3 nM, 10 nM) did not increase CaSF as compared with 0 nM ISO, while 30 nM, 100 nM ISO significantly increased CaSF as compared with 0 nM ISO. Each group included 20–30 cells. At least 4 cells were evaluated for each preparation. The bars indicate the means ± SE. CaSF, frequency of Ca2+ sparks; ISO, isoproterenol (TIF) [file pone.0163250.s002.tif]
